# Applying the consolidated framework for implementation research to identify barriers affecting implementation of an online frailty tool into primary health care: a qualitative study

**DOI:** 10.1186/s12913-018-3163-1

**Published:** 2018-05-31

**Authors:** Grace Warner, Beverley Lawson, Tara Sampalli, Fred Burge, Rick Gibson, Stephanie Wood

**Affiliations:** 1 Dalhousie University, Halifax, NS Canada; 2 Healthy Populations Institute, Halifax, NS Canada; 3 Building Research for Integrated Primary Healthcare (BRIC NS), Nova Scotia Primary & Integrated Health Care Innovations Network, Halifax, NS Canada; 4 Primary Care Research Group, Dalhousie Family Medicine, Halifax, NS Canada; 5 Primary Health Care, Nova Scotia Health Authority, Halifax, NS Canada; 6 Department of Family Practice, Nova Scotia Health Authority, Halifax, NS Canada

**Keywords:** Frail elderly, Primary health care, Patient care planning, Web-based frailty portal

## Abstract

**Background:**

Frailty is associated with multi-system deterioration, and typically increases susceptibility to adverse events such as falls. Frailty can be better managed with early screening and intervention, ideally conducted in primary health care (PHC) settings. This study used the Consolidated Framework for Implementation Research (CFIR) as an evaluation framework during the second stage piloting of a novel web-based tool called the Frailty Portal, developed to aid in the screening, identification, and care planning of frail patients in community PHC.

**Methods:**

This qualitative study conducted semi-structured key informant interviews with a purposive sample of PHC providers (family physicians, nurse practitioners) and key PHC stakeholders who were administrators, decision makers and staff. The CFIR was used to guide data collection and analysis. Framework Analysis was used to determine the relevance of the CFIR constructs to implementing the Frailty Portal.

**Results:**

A total of 17 interviews were conducted. The CFIR-inspired interview questions helped clarify critical aspects of implementation that need to be addressed at multiple levels if the Frailty Portal is to be successfully implemented in PHC. Finding were organized into three themes 1) PHC Practice Context, 2) Intervention attributes affecting implementation, and 3) Targeting providers with frail patients. At the intervention level the Frailty Portal was viewed positively, despite the multi-level challenges to implementing it in PHC practice settings. Provider participants perceived high opportunity costs to using the Frailty Portal due to changes they needed to make to their practice routines. However, those who had older patients, took the time to learn how to use the Frailty Portal, and created processes for sharing tasks with other PHC personnel become proficient at using the Frailty Portal.

**Conclusions:**

Structuring our evaluation around the CFIR was instrumental in identifying multi-level factors that will affect large-scale adoption of the Frailty Portal in PHC practices. Incorporating CFIR constructs into evaluation instruments can flag factors likely to impede future implementation and impact the effectiveness of innovative practices. Future research is encouraged to identify how best to facilitate changes in PHC practices to address frailty and to use implementation frameworks that honor the complexity of implementing innovations in PHC.

## Background

While the word frail is common to many health care settings, the actual diagnosis of frailty is complex due to varied presentations and causes [1]. Frailty is often defined as a physiologic syndrome characterized by decreased reserve and resistance to stressors, resulting from a cumulative decline across multiple physiologic systems; causing vulnerability to adverse outcomes [2]. It is generally associated with multi-system (e.g., mobility, cognition, function, endurance) deterioration, and typically impacts the geriatric population. Persons experiencing frailty are highly susceptible to adverse events such as falls, hospitalization, disability, dependence, placement in long-term care facilities and death [2, 3]. Since frailty is a robust marker of vulnerability it is important to accurately identify those who are frail, so they can be better managed with early identification and appropriate interventions that reduce adverse events [4, 5].

Given the majority of frail persons live in the community, and providers who work in primary health care (PHC) regularly encounter frail persons in their daily clinical work [6], strengthening PHC for frail adults is crucial. Identification of frail patients in PHC is a pro-active approach to providing care [7] that can improve patient understanding of their overall health and engage them and their family in the decision-making process with their health provider regarding preventive strategies [8, 9]. Routine identification of frailty offers opportunities for targeted care including the application of newly developed clinical practice guidelines for frailty [10, 11]. However, routine identification and measurement of frailty is not part of standard care and is only now emerging as a concept for primary care [12, 13].

To enable frailty screening and interventions to effectively and consistently occur in PHC, providers need appropriate tools for identifying frailty [14]. Recent advances in technology have enabled easy, timely and relevant access and application of tools at the point of care [15]. The use of technology has evolved as a practical and feasible option for embedding tools to support evidence-informed care, increasing the application of knowledge into practice. To assess and address the needs of frail individuals in the community a web-based tool called the Frailty Portal was created to be used in PHC practices.

### Consolidated framework for implementation research

As part of the evaluation of the Frailty Portal, barriers and facilitators to implementation were assessed using the Consolidated Framework for Implementation Research (CFIR) as an evaluation framework. The CFIR was chosen because it is a relatively new framework, that has synthesized prior research evidence representing a spectrum of disciplines into one consolidated framework with multiple constructs to create a typology of what affects successful implementation of an intervention [16]. It has 39 constructs organized into five major domains found to influence the successful implementation of innovative programs. The domains assess i) Intervention characteristics (eight constructs), ii) Outer setting (four constructs), iii) Inner setting (14 constructs), iv) Characteristics of individuals (five constructs) and, v) Process (eight constructs) [17]. Table 1 lists all the CFIR domains and constructs.

**Table 1:** CFIR domains and associated constructs

1. Intervention Characteristics	4. Characteristics of Individuals
○ Intervention source	○ Knowledge and beliefs about the intervention
○ Evidence strength and quality	○ Self-efficacy
○ Relative advantage	○ Individual stage of change
○ Adaptability	○ Other personal attributes
○ Trialability	
○ Complexity	
○ Design quality and packaging	
○ Cost	
2. Outer Setting	5. Process
○ Patient needs and resources	○ Planning
○ Cosmopolitanism	○ Engaging
○ Peer pressure	○ Executing
○ External policy and incentives	○ Reflecting and Evaluation
3. Inner Setting	
○ Structural characteristics	
○ Networks and communication	
○ Culture	
○ Implementation climate	
○ Readiness for implementation	

A recent systematic review by Kirk et al. (2016) [17] examined how studies currently use the CFIR and how it might be used in the future. They found the CFIR had most often been employed in doing a post-hoc analysis on what facilitated or hindered implementation. It has been less frequently used at the pre-implementation stage to identify barriers and facilitators that could affect future implementation [18, 19]. The aim of the study was to use the CFIR as an evaluation framework at the pre-implementation stage to clarify critical barriers and facilitators to implementation that need to be addressed at multiple levels if the Frailty Portal is to be successfully implemented in PHC practices.

## Methods

### Development of the frailty portal

The Nova Scotia Health Authority PHC (NSHA-PHC) initiated a Frailty Strategy in 2012 to achieve its goal of improving care for its frail population. An objective of the strategy was to assess and address Frailty in PHC. To achieve this objective NSHA-PHC created a web-based tool called the Frailty Portal in collaboration with community partners from other health care sectors such as home care and geriatrics, and community volunteer agencies that addressed the needs of older adults. The Frailty Portal has two essential components: 1. an assessment phase, and 2. practice visit goals. Within the assessment phase the provider is to first identify conceivably frail patients and then screen those identified to determine their level of frailty using a web-based version of the Frailty Assessment for Careplanning Tool (FACT) [20], which is a modification of the Clinical Frailty Scale [21]. The FACT assesses essential domains that contribute to frailty (cognition, mobility, function and social circumstances) and provides a score to measure the patient’s frailty level (thriving to terminally ill). Based on this frailty level, the second component of the Frailty Portal provides practical visit goals, tailored to the patient’s identified frailty level, for use in care plan development and links to relevant resources for providers, patients and caregivers. Additional detail about the intervention has been previously published [22].

The assessment component was initially piloted in 2014 among a limited number of PHC physicians who were asked to provide their impressions of the required steps for identification and screening of patients using the FACT as well as the overall usability of the web-based tool within community PHC care practice. Based on these suggestions modifications were made to the web-based interface to improve usability of the assessment tool and maneuverability within the site. As well a second component was added that provided practical visit goals and a toolkit of currently available resources.

In this article we focus on data gathered during the second piloting of the Frailty Portal in 2015–2016 which followed modifications from the first pilot and the addition of the second component. For this second pilot a broader group of PHC providers that included physicians and nurse practitioners were asked to take part. As part of the second pilot a formal half-day education workshop offering detailed information about identifying frailty with hands-on learning using the Frailty Portal tool was provided.

### Setting

In Canada, PHC is partially funded through public funds that are allocated by the health authorities within each province. The outer setting for this study is NSHA-PHC, which encompasses urban, sub-urban, and rural service locations. Diverse support services are available in the different locations. At the level of the practice setting, the CFIR inner setting domain, PHC includes both team-based and individual practices that are remunerated through various payment plans, the majority through fee-for-service. In some practice settings PHC providers, the individuals involved in implementing the Frailty Portal, were employees of the NSHA while others were from private practices Although the work of most community-based family physicians is not under the direct responsibility of the NSHA; the NSHA directly engages and supports family practices in their work and involves them in health authority driven initiatives. This study was part of a health authority initiative.

The NSHA research ethics board reviewed our protocol and procedures, the study was considered to be a program quality initiative that did not require individual consent from participants. Although consent was not deemed necessary all participants were informed that no personal information would be shared in our summaries, but they would be labeled by PHC role. Also, that any personal information they shared with us would remain confidential and necessary precautions would be taken to ensure their data was kept in a secure password-protected location.

### Participants

This qualitative descriptive study was one part of a larger convergent mixed methods study. The protocol for the entire study is described in a previous publication [22]. Semi-structured key informant interviews were conducted with a purposive sample of PHC providers and key PHC stakeholders who were administrators, staff, and decision makers. Decision makers were higher level administrators who had the authority to make policy and funding decisions in PHC-NSHA. Potential participants were identified by NSHA then purposively sampled to provide different perspectives on the history, development and implementation of the Frailty Portal.

### Interview guides

The initial interview guides were based on sample interviews available on http://cfirguide.org/ then tailored to gather specific information about the Frailty Portal intervention [18]. Damschroder et al. (2009) [18] recommend that implementation researchers try to pre-identify CFIR constructs they will assess based on the relevancy to the study, then determine what level each construct should be measured. They also recommend that researchers report their decisions and rationales for choosing certain constructs, along with findings for each construct that is ultimately selected. For this study, the CFIR domains aligned with the following entities: Intervention characteristics (of the Frailty Portal); Outer setting (NSHA-PHC); Inner setting (PHC practices); Characteristics of individuals (PHC providers who piloted the Frailty Portal); and, Process (aspects of developing, delivering and evaluating the Frailty Portal). There were CFIR constructs within these domains that the literature suggested might be less salient to Frailty Portal implementation success; however, the research team decided to probe all the CFIR domains and constructs in the interviews because there was no definitive evidence.

The same semi-structured interview guide was used for all stakeholders; however, because stakeholders had varying backgrounds they were first queried whether they had specific knowledge related to a section to determine if the questions were relevant. The interview guide was divided into sections that covered 1) background information on the initial development and first piloting of the Frailty Portal, and 2) an evaluation of the Frailty Portal process and tool based on experiences during the second piloting. The first section provided the researchers with supplemental information on the development of the Frailty Portal. The second section was the focus of our study. If participants felt they could not contribute information to particular questions, they were skipped. Interviews were completed in-person at a location convenient to the interviewee, or by telephone if distance was prohibitive. All interviews were conducted by the first author, who was an independent qualitative researcher outside of the NSHA trained in qualitative interviewing and analysis. The interviews were recorded using a digital audio recording device for ease of transcription and review. Data were transferred from the device following each interview and transcribed verbatim by an experienced transcriptionist. Following review of the interview transcription the interviews were uploaded into NVivo For Mac 11.2.2 qualitative software for analysis. All interviews were de-identified; a code was given to each interview and personal identifiers were stripped from the data.

### Analysis

Descriptive qualitative research, using a Framework Analysis approach [23, 24] was used during the study to determine the relevance of the CFIR constructs. Qualitative description is used to describe rather than interpret phenomenon through an identified theoretical framework, such as phenomenology or grounded theory [25]. In qualitative description, the researcher collects data to understand the area of study then describes this data using everyday terms as they relate to the event or area of study. Content Analysis, the process of making sense of the meanings in the data, was also used during our thematic analysis [26].

The Framework Analysis followed the five-step process outlined by Richie and Spencer (1994); 1) familiarization, 2) identifying a thematic framework, 3) indexing, 4) charting, and 5) mapping/interpretation. The analysis was an ongoing iterative process. A research assistant worked with the first and second author to conduct multiple reviews of the transcripts and tapes to familiarize (Step 1) themselves with the data and identify initial themes that were reflexive and interactive. Analysis was initiated as soon as the first interview was completed and continued concurrently with data collection to help determine when new information was no longer being generated from interviews. Although the team identified the CFIR as the apriori framework, additional codes emerged during the familiarization process to develop a thematic framework (Step 2) that reflected the language and experiences of participants. The codes also reflected relevant CFIR constructs across the five domains and were indexed (Step 3) to sections of the transcripts using NVivo. An audit trail was used to document our decision-making process. Sections of the transcripts were charted into themes using Excel (Step 4). First they were organized by CFIR domains and constructs, then re-framed to better reflect descriptions from participants. All three analysts reviewed the codes and associated themes multiple times to check for potential biases, to ensure they reflected participants’ words, and improve the credibility of their interpretation (Step 5) of the interviews. Additional interviews were added with physicians when new themes emerged, to ensure saturation was reached. Initial findings were shared with a group of participants to help with interpretation and generate meaning from the data. To ensure the data was collected, analyzed and interpreted accurately, so it conveyed the experiences of participants, processes associated with trustworthiness were enacted such as member checking and reflexivity [27].

## Results

A total of 17 interviews were conducted. PHC stakeholder participants (noted as SH in the quotes) included decision makers (*n* = 2), health authority administrators (*n* = 4), and staff (*n* = 2). PHC providers interviewed (noted as HP in the quotes) were family physicians (*n* = 6) and nurse practitioners (*n* = 3). The interviews lasted from 40 min to 1.5 h. Although we considered presenting our findings by CFIR domains, our thematic framework indicated the domains overlapped. The complexity of the intervention and implementation processes made it difficult to separate key findings by domain. As such our findings are organized into three themes that reflected participants’ experiences with the Frailty Portal but are informed by the CFIR framework; 1) PHC Practice Context, 2) Intervention attributes affecting implementation, and 3) Targeting providers with frail patients. Quotes are provided to illustrate each theme. The CFIR constructs identified in the themes are listed in Table 2.

**Table 2:** CFiR Domains and Constructs Associated with Qualitative Themes

Theme	CFIR Domain	CFIR constructs
1: PHC Practice Context	Outer Setting	Patient needs and resources, external policy, incentives, peer pressure, cosmopolitanism
	Inner Setting	Compatibility, networks, communications, learning climate, culture
	Characteristics of individuals	Self-efficacy
	Intervention	Costs (opportunity).
	Process	Planning
2: Intervention attributes that affected implementation.	Inner Setting	Access to knowledge and information
	Characteristics of Individuals	Knowledge & Beliefs about the Intervention, self-efficacy
	Intervention	Evidence strength, complexity, adaptability, design quality & packaging; cost (opportunity).
	Process	Planning, engaging, champions
3: The importance of targeting providers with frail patients.	Outer setting	Patients’ needs and resources
	Characteristics of individuals	Knowledge and beliefs about the intervention, individual stage of change
	Intervention	Costs (opportunity)

### Theme 1: PHC practice context

The PHC Practice Context is affected by several CFIR domains. Most providers identified constraints at the level of the health authority that affected how they set up practice routines, thus identifying outer setting factors such as resources and external policies that put pressure on providers to see a certain number of patients within a given timeframe. Family physicians felt pressured to see a patient every 15–20 min, this was not conducive to completing the Frailty Portal which took more time. In a fee-for-service environment it would have been helpful to pair the intervention with a specific payment mechanism that would compensate providers for longer assessment visits. In the CFIR this would be classified as an incentive at the level of the outer setting.

If you happen to have two physicians doing a couple of frailty assessments taking 45 min each, that drastically reduces your patient capacity. HP3

…we need more resources to be able to really roll it out [to other practices]... HP7

…Is there a [fee] code for the extra time? HP8 (medical lead)

Outer setting constraints made the intervention incompatible with routines used within the inner setting of PHC practices to see patients. Integrating the Frailty Portal into practice routines required time, which was an opportunity cost to the physician. Opportunity costs refers to a situation where the physician loses the potential gain from seeing another patient because they have used that time to complete the Frailty Portal. Providers who took time to complete the Frailty Portal or learned how to integrate it into their practice routines became proficient at using the Frailty Portal, had increased self-efficacy with the intervention, and were likely to use the tool more regularly.

…two to five visits with somebody in order to get through the assessment and planning is not a typical structure [for seeing patients]. …. HP4

In contrast, the practice context of the PHC nurse practitioners was different from physicians. The nurse practitioners who were interviewed felt the Frailty Portal was compatible with their practice as it aligned with their capacity for longer appointment times and scope of practice regarding chronic condition management (e.g. frailty). As such they were better able to fit the use of the Frailty Portal into their practice routines, however, they still had to ensure other providers in the practice were supportive of their allocating time to implement the Frailty Portal instead of seeing additional patients. This required communication and negotiations with other individuals in the practice.

….the nurse practitioners that are using the Portal. They’ve got a little bit more flexibility… for them to bring a patient in for half an hour, 45 min to do a frailty assessment, no big deal... SH2

It makes perfect sense and it fits right in keeping with what we’re [nurse practitioners] doing…The problem is the time pressure. And it’s not always accepted by the general culture of the clinic. HP6

The PHC practice setting is unique; family physicians do not work in a typical “organizational” structure. They are often independent businesses that are not networked with other practices in the health authority. Not working within a typical organizational structure lowered the effectiveness of using peer pressure or organizational culture to stimulate change. There was no group culture to support change in PHC practices. PHC physicians who worked in larger teams with access to nurse practitioners, or support staff, could share the workload and reduce the time needed to implement the Frailty Portal. Teams were encouraged to work together to develop a plan for identifying potentially frail patients in advance, and schedule appointments for assessing and addressing frailty. These teams were more successful at implementing the Frailty Portal.

…the plan had been to send out [a report to provide an incentive to providers saying]… “this is how many assessments have been done by your group, here’s the level of frailty, here’s the average age”,…that really didn’t seem to be an incentive for folks. SH6

If I had her [family practice nurse] probably book even an hour of her time…to do a lot of the [Frailty Portal] questions and getting the information, and… reviewing some of the [Frailty Portal] care planning with them, [it] would be a good use of time. HP11

### Theme 2: Intervention attributes that affected implementation

Providers commented positively on the half-day training session for the Frailty Portal and felt it was informative. The session was co-led by providers viewed as leaders in their practice community who demonstrated their support for the intervention. The support from practice leaders and the health authority satisfied attendees that the Frailty Portal was evidence-based. However, providers felt the training would have benefited from a follow-up session shortly after the initial training. This follow-up session could address problems that occurred when the Provider first attempted to use the Frailty Portal in their practice setting. Most of the comments were around the difficulty of implementing processes in their practice to do all the steps associated with the Frailty Portal.

The training was excellent. I would have liked more around the planning part because that’s where I really feel like I fell short. HP6

… a two-part [training] session where you are introduced to it [Frailty Portal], you go ahead and try it, and then you’re scheduled to come back…would have been helpful. HP1

Providers felt the Frailty Portal was attractive and well designed. The Frailty Portal functioned outside of the existing system for documenting patient medical information, which for many practices was the electronic medical record (EMR). The Frailty Portal required logging into a secure web-based system with firewalls created to ensure patient privacy. Some providers had challenges accessing the site due to these security features. They had trouble with passwords expiring and not remembering how to reset them. Solving the problem required a real-time phone conversation with a help desk. Often the provider made their first attempt at accessing the Frailty Portal post training during a patient encounter. If there was a problem logging into the system (e.g., web page not displaying, passwords expired) immediate assistance was needed, the providers often didn’t remember who to call or where to reach out. If they did not get assistance quickly, the provider was frustrated and likely abandoned using the Frailty Portal all together. Data from the Frailty Portal was saved separately from the EMR; therefore, it needed to be entered into the EMR at a later time. Administrators were aware of this problem and were actively working to identify ways to integrate the Frailty Portal with existing EMR provider software.

I attempted to get into the Portal a number of times when I had a client in front of me, … and I had difficult logging on. I couldn’t figure out what was going on. HP1

…they feel that what they enter here [in the Frailty Portal] is redundant with what they’re going to enter in their own EMR…. we’re looking at, is there a way that we can send the results… directly to their EMR? SH1

I had a lot of difficulty with logging into the Portal…. she was going to call me back and help me with the username and password. But I never received anything…until I called them back. HP10

Within the FACT, the frailty assessment tool embedded in the Frailty Portal, was a separate collateral form which providers were to ask family members to complete. This information was to confirm providers’ assessments of frailty. However, providers felt challenged scheduling patient appointments that included family members. This often resulted in the form not being completed. Instead some providers used their own judgement rather than confirming their frailty assessment with the family; others realized the importance of getting family input. One provider wondered about privacy concerns if they asked family members about the patient.

You know, what does your patient think about you asking their family members about them? HP3

… where we’re trying to assess for frailty, it’s not typical that a caregiver would be part of that… HP4

I’m suspecting that it’s probably better that you do ask somebody in the family who sees them the other 364 days of the year what’s really going on. HP8 (medical lead)

The last stage of the Frailty Portal provided suggestions for care plans based on the patient’s frailty level. The care plans occasionally involved referrals to community organizations. When providers had limited knowledge of an organization it was difficult for them to quickly judge the relevance and appropriateness of the referral. Although the Frailty Portal referred providers to the organization’s website for information about the organization, this learning process was time consuming.

…making sure that you’ve referred to all the appropriate places…really puts it all together and it gives you an overall picture of…what you need to do for clients. HP9

…I like some of the links and the resources…But to be able to work through that whole care plan…that you’ve completed that assessment for that person, that’s a big ask...HP2

A final challenge identified was using the Frailty Portal over multiple visits. If a patient’s condition was to change over time the provider may be required to re-assess and develop new care plans without finishing the first one. Some providers mentioned they never completed the “record” for their patient. This lowered their self-efficacy for using the tool.

So… I don’t really get it finished because their care plan is so complex that it’s overwhelming. …I get lost in trying to keep it going. HP11

Basically, it’s when should the chart be closed? SH1

When interview participants were asked, “*On a scale of 1-10, with one being very easy and 10 being nearly impossible, how difficult was the Frailty Portal Initiative to implement?”*

Administrators and providers commonly rated the difficulty, or complexity, of the intervention between 6 and 7. The reason for the high rating was usually due to the multiple Frailty Portal components and the necessary changes that needed to be made to practice routines to incorporate it into their practice.

The first nine screens are a one – very easy. It’s that last screen that’s challenging because it’s just information overload. HP6

Maybe six, seven because of the obstacles… for the last care plan page, that I think it is very difficult, very time-consuming and needs training. HP2

### Theme 3: The importance of targeting providers with frail patients

The NSHA-PHC decision makers had identified frailty as an important condition to address their patient population’s needs so health system changes could be resourced and implemented to improve current quality of care and reduce long term costs. They identified PHC providers as the first point of contact for frail patients and felt early identification of frailty would benefit the health system.

We identified that we had a growing problem with our frailty populations.. SH3

So… it sort of fell in a bucket of things we were trying to do…so that we can improve the care of the population and help family doctors do their job better or more efficiently. HP8 (medical lead)

The administrators made the decision to pilot the Frailty Portal with providers who cared for a wide range of patient populations in their practices. The level of provider support for the Frailty Portal varied depending on their patient population. Providers who had a more geriatric patient population believed in the value of using the Frailty Portal and viewed it positively. However, provider interviews showed there was limited motivation for changing current practices, or individual stage of change, to implement the Frailty Portal. The current culture in PHC practice settings did not view the Frailty Portal as a priority compared with other daily tasks and activities they needed to perform.

I’m not saying I shouldn’t do it and it’s not the right thing to do for that patient but you’ve now created another mammoth load of work for me… HP4

I think we could better have tailored…which practices have the patient population to use for this [pilot]. On the other hand, it would be a little bit like preaching to the converted …And it really is the physicians who aren’t as geriatric savvy who could benefit from this tool and using it. HP3

I just think we need to think about why we’re doing it and…the benefits that are coming from investing the time in doing that …you know, there are opportunity costs. HP1

## Discussion

The aim of the study was to use the CFIR as an evaluation framework at the pre-implementation stage of a web-based tool called the Frailty Portal to clarify critical barriers and facilitators to implementation that need to be addressed at multiple levels if the Frailty Portal is to be successfully implemented in PHC practices. Although some of the obstacles to implementation were expected, the CFIR-inspired interview questions helped clarify critical aspects of implementation that need to be addressed if the Frailty Portal is to be successfully integrated into PHC practices.

At the intervention level the Frailty Portal was viewed positively, despite the multi-level challenges to implementing it in their practice settings. Similar to findings in other implementation literature, interventions must be tailored to fit within different practice contexts, and it is important for providers to believe there is a need for the intervention [28]. The study identified key intervention characteristics that can be modified to reduce the complexity, increase its adaptability, and reduce provider opportunity costs. For some providers, only slight modifications are needed such as removing barriers to logging onto the server where the tool is housed or providing direction on how to integrate the Frailty Portal into practice routines.

At the provider level, study participants perceived a high opportunity cost to using the Frailty Portal resulting in an inability to see other patients. These opportunity costs were less if their scope of practice included time to address prevention or their practices had a high proportion of older patients. Although it is likely most providers will become faster at completing the Frailty Portal with practice, and their self-efficacy should increase, they first need to commit time to becoming proficient.

Organizational changes (inner setting) that facilitate sharing administrative and assessment responsibilities within the team could reduce providers’ opportunity costs. For example, administrative staff can pre-identify frail patients and set up frailty-specific appointments with patients and their family members. Furthermore, creating networks between PHC practices and trusted community programs could increase team members’ confidence referring patients outside of the health care system. However, the larger issue is the need to cultivate a practice culture that values the need to screen for and address frailty. The findings suggest, and the literature confirms [29] that until that shift in culture occurs it would be beneficial to concentrate on providers who are more likely to use the Frailty Portal, leaving those who are less ready for change to do so at a later time.

In conjunction with practice level changes, external policies, incentives and training should be considered by the appropriate external bodies (outer setting) such as the provincial health authorities in Canada. Incentives may include creating billing codes to provide monetary compensation for the additional time necessary to access and develop care plans for frail patients. Training and education may also improve implementation. Education on the importance of assessing frailty could improve beliefs about the need to assess frailty, and training on how to distribute Frailty Portal tasks within the team should increase self-efficacy for implementing the Frailty Portal. Other research has shown these types of incentives facilitate uptake [28]. Most importantly, strategies need to be developed for how best to communicate with PHC providers. The Frailty Portal training staff tried several communication strategies to provide helpful suggestions on how best to integrate the Frailty Portal into providers’ practice routines, but they were unsuccessful due to providers’ busy schedules and lack of dedicated time for training and education. This is a major barrier to implementing innovative tools such as the Frailty Portal.

Our findings may be limited by our choice of participants. Although the number of interviews was small, participants included those who had both experience using the Frailty Portal in a PHC setting and stakeholders who created and helped implement the Frailty Portal. Additional interviews were added when new themes emerged to ensure themes reflected participants’ experiences. Furthermore, findings were presented to participants and other PHC stakeholders to confirm researchers’ interpretation of the interviews. Presentations also helped clarify aspects of the practice setting that need to be considered when NSHA-PHC initiates implementation of the Frailty Portal in other PHC practices.

Prior research has used the CFIR to identify distinguishing constructs between high and low implementation success [30]. This was not the intent of our study, but one construct found to be associated with successful implementation was also identified across all three of our themes; opportunity costs. Our findings highlight the interconnectivity of the constructs. High opportunity costs relate to the providers’ perceptions that the intervention takes too much time to implement. However, this perception is affected by other domains and constructs also identified in the study. Opportunity costs are affected by individual providers’ beliefs regarding the importance of assessing frailty, the inner setting construct identifying the importance of having a learning climate in the practice setting, and outer setting constructs in control of the health authority such as billing codes. The CFIR was useful for not only identifying constructs, but for acknowledging the relationships between constructs.

Similar to other implementation science frameworks [31, 32], the CFIR is better suited to assessing implementation in facilities where individuals work within a clear organizational structure. Within community PHC practices, CFIR constructs such as organizational networking and communication and peer pressure did not facilitate implementation. PHC providers are often independent practitioners, so group pressure is virtually nonexistent. The field of implementation science has largely developed frameworks for institutional settings, rather than community settings. Despite this drawback, the CFIR framework was easily adapted for PHC settings and helpful for identifying key factors important to successful implementation.

Finally, there are important reasons for PHC providers to identify and treat frailty; the rising number of older adults [33], the parallel increase in frailty [34], and the need to initiate care proactively before an adverse event occurs stimulating further decline leading to hospitalizations and possibly long- term institutionalization [4, 5]. PHC providers in our study confirmed they felt it was important to assess and address frailty in their community-based practices. Unfortunately, they also found it challenging to implement the Frailty Portal into practice routines. For some it may have been due to the complexity of the Frailty Portal tool itself, for others it was due to difficulty accessing the online platform. For most providers using the Frailty Portal required a significant time commitment to assess frailty then enact resultant care plans. A more easily accessible tool that is less time consuming to administer such as the Clinical Frailty Scale [21] would likely have less barriers to implementation; however, it does not link to an actionable care plan so it may not facilitate better patient care. A better option is to integrate the Frailty Portal into the EMR, and to share frailty assessment and care plan development with appropriate PHC team members, depending on their scope of practice, to reduce individual burden and improve quality of care.

## Conclusions

Our study supports prior recommendations for using CFIR [19, 35], and more broadly implementation science frameworks [36, 37], to facilitate implementation of complex interventions. Despite the fact that the CFIR is biased toward institutional care and would benefit from modifications to better capture attributes unique to PHC, structuring our evaluation around the CFIR was instrumental in identifying multi-level factors that will affect large-scale adoption of the Frailty Portal in PHC practices. The implementation of the Frailty Portal within community PHC practices is representative of a complex, transformation, health system intervention. Not only are the needs of the patient and their caregiver multifaceted and complex, but the context of primary care practice is as well.

To successfully integrate the Frailty Portal into everyday routines of PHC providers barriers need to be addressed at multiple levels. At the NSHA-PHC outer setting level linking it to the EMR will facilitate providers’ initial use of the tool and establishing appropriate fee structures that compensate providers for the additional time necessary to assess and address frailty will sustain its long-term use. Furthermore, at the PHC practice level it is better to initially implement the Frailty Portal in primarily geriatric practices. Also practices that have team members who can share administrative and assessment responsibilities with the provider will reduce individual opportunity costs.

It is beneficial to identify barriers at the pre-implementation stage so they can be addressed early. One barrier that will hopefully reduce over time is the development of a PHC practice culture that values the need to screen for and address frailty, and considers it part of best practices in PHC. Future research is encouraged to identify how best to facilitate changes in PHC practices to address frailty and to use models that honor the complexity of implementing innovations that can improve care for frail patients and their caregivers in the community.

## Abbreviations

CFIR: Consolidated Framework for Implementation Research framework
EMR: Electronic medical record
FACT: Frailty Assessment for Careplanning Tool
HP: Health providers
NSHA-PHC: Nova Scotia Health Authority PHC
PHC: Primary health care
SH: Stakeholder
